# Editorial: The role of extracellular vesicles in diseases: Shedding light on their role in cell-to-cell communication

**DOI:** 10.3389/fgene.2023.1123822

**Published:** 2023-01-24

**Authors:** Gabriela Repiska, Rossella Crescitelli, Taral R. Lunavat, Carolina Soekmadji, William C. Cho

**Affiliations:** ^1^ Institute of Physiology, Faculty of Medicine, Comenius University in Bratislava, Bratislava, Slovakia; ^2^ Sahlgrenska Center for Cancer Research and Wallenberg Centre for Molecular and Translational Medicine, Department of Surgery, Institute of Clinical Sciences, Sahlgrenska Academy, University of Gothenburg, Gothenburg, Sweden; ^3^ Department of Neurology, Molecular Neurogenetics Unit-West, Massachusetts General Hospital, Harvard Medical School, Charlestown, MA, United States; ^4^ Department of Biomedicine, University of Bergen, Bergen, Norway; ^5^ School of Biomedical Sciences, Faculty of Medicine, University of Queensland, Brisbane, QLD, Australia; ^6^ Department of Clinical Oncology, Queen Elizabeth Hospital, Kowloon, Hong Kong SAR, China

**Keywords:** extracellular vesicles, cancer, cancer immunology, liver transplantation, protozoan infection, ciliary EVs

Research on extracellular vesicles (EVs) has grown exponentially in the biology and biomedical sciences (Fais et al., 2016; Azparren-Angulo et al., 2021). Classical EVs, such as exosomes and ectosomes that include small-sized EVs (such as small ectosomes), medium-sized microvesicles and larger-sized apoptotic vesicles, have been studied in depth on their role in health and diseases (Carpintero-Fernandez et al., 2017; Buzas, 2022). Additionally, a recent understanding of EV subtypes has been supported by emerging technologies and new tools developed to deeply separate EV subpopulations (Thery et al., 2018). Part of the research work in the EV field focuses on the analysis of the characteristics, biogenesis and physiological roles of these newly identified secreted vesicles. There is considerable interest in the potential use of EVs as clinical biomarkers, and therapeutic targets for diseases since EVs are actively released from cells into the extracellular space as well as into body fluids (Tenchov et al., 2022). The cargo of EVs is composed of proteins, lipids, and different types of nucleic acids and it is influenced by the origin of the cell and its growth conditions. Moreover, EVs can be isolated from a variety of biological fluids (urine, milk, blood, and saliva) (Faur et al., 2022; Rashidi et al., 2022; Schaack et al., 2022; Yoon et al., 2022). All these EV characteristics make them a promising source of disease biomarkers. This Research Topic of articles explores how EVs may play a role in pathological conditions, including cancer, rejection after organ transplant, protozoan infections, and cilia-related pathologies.

Procaryotic cells, such as parasites, can transmit information to their hosts by secreting EVs that modulate their immune responses. In the review article by Wang et al., the roles of EVs secreted by different kinds of protozoa responsible for parasitic diseases were discussed. EVs are involved in developing Chagas disease, leishmaniasis, giardiasis, trichomoniasis, amoebiasis, malaria, and neosporosis. Moreover, EVs are involved in the immune escape of parasites, and the transmission of parasitic infections (Schorey et al., 2015). Wang et al. concluded that EVs have a complex effect on the host, such as regulating the expression of inflammatory factors in macrophages. It is important to understand how EVs affect parasites in order to develop therapies against parasitic diseases.

A study published by Sass et al. in cancer immunology reported that soluble and EV-associated cytokines showed distinct symptom profiles in breast cancer patients before surgery. According to their findings, there may be an association between a higher symptom burden and inflammation, that in turn affects EV content. Cancer patients may experience co-occurring symptoms as a result of discrepancies in the production of cytokines. One of the highlights of their findings was the positive association between EV and soluble pro-inflammatory cytokines and fatigue, regardless of the age group or symptom profile. Furthermore, within the older cohort, the concentrations of EV-associated granulocyte-macrophage colony-stimulating factor and IL-2 were higher in patients with high fatigue and low pain symptom profiles than in patients with low symptom profiles. By clarifying the mechanisms that cause distinct symptom class profiles, intervention trials may be informed, and the approaches for precision medicine can be developed. It is important to note that the additive effect of aging and cancer may need to be explored using a larger sample size and longitudinal designs.

In metastatic cancer, Su et al. reported the role of the long non-coding RNAs (lncRNAs) in tumor development and immune response. Their results show that exosome-related lncRNAs can provide a predictive signature for liver cancer. The expression of five lncRNAs (MKLN1-AS, TMCC1-AS1, AL031985.3, LINC01138, and AC099850.3) was linked to a poor prognosis for liver cancer patients, providing a means to predict risk in liver cancer. They discovered that this model is closely related to the immune cell microenvironment, opening the avenue for a potential direction for future cancer immunotherapy research.

Wang et al. discussed the potential use of EVs as biomarkers for acute rejection (AR) in patients subjected to liver transplantation. As a common and serious complication of transplantation, AR poses a challenge for diagnosis since there are no biomarkers, and the only available assay requires invasive techniques. Based on an analysis of microRNAs isolated from the serum of AR patients, the authors identified 614 microRNAs that were significantly altered in diseased patients compared with patients without any symptoms of rejection. These differentially expressed microRNAs target genes involved primarily in ubiquitin-mediated proteolysis, lysosomes, and protein processing in the endoplasmic reticulum. These findings suggest that microRNAs derived from EVs may be useful as biomarkers of AR in liver transplant recipients.

Zhang et al. summarized the characteristics and the role of EVs in bone remodeling processes, including bone tumor development, vascular skeletal muscle injury, spinal cord injury, degenerative disc disease, cartilage degeneration, osteoarthritis, necrosis of the femoral head, and osteoporosis as models of bone diseases. EVs are ideal candidates to be utilized in drug delivery systems to treat orthopedic diseases. It should be noted, however, that most EV research is at the preclinical stage. It is imperative to develop appropriate methods of isolating, purifying, and implementing EVs for the diagnosis, monitoring, and treatment of orthopedic diseases.

In the last decade, a new type of EV has been discovered within the ciliary organelles. Primary cilia are microtubule-based organelles located on the surface of most cells that are responsible for controlling homeostasis (Mohieldin et al., 2021; Wang and Barr, 2016). Vinay and Belleanee evaluated the current state of knowledge regarding ciliary EVs. This paper examined the characteristics, functions, and mechanisms underlying the release of ciliary EVs from mammalian ciliated cells. Ciliary EVs have been the subject of only a limited number of research studies, and further investigations are needed to determine if they can be used as a biomarker of diseases related to cilia.

As a highly interesting and rapidly evolving field, EV research could contribute significantly to modern medicine by providing information about physiological states and early pathological changes as prognostic and diagnostic markers and as smart nanoscale therapeutics. EVs have the potential to be an effective way of treating diseases that have been exceedingly difficult to treat up until now.
